# Foodborne Diseases Active Surveillance Network (FoodNet).

**DOI:** 10.3201/eid0304.970428

**Published:** 1997


### Foodborne Diseases Active Surveillance Network (FoodNet)

The Foodborne Diseases Active Surveillance Network (FoodNet) is the foodborne disease component of the Emerging Infections Program (EIP) of the Centers for Disease Control and Prevention (CDC). A collaborative project of CDC, the seven EIP sites, the U.S. Department of Agriculture (USDA), and the U.S. Food and Drug Administration (FDA), FoodNet consists of active surveillance for foodborne diseases and related epidemiologic studies designed to help public health officials better understand the epidemiology of foodborne diseases in the United States. FoodNet was established in 1995 in five locations: Minnesota, Oregon, and selected counties in Georgia, California, and Connecticut. The total population of these sites, or catchment areas, is 14.7 million, or 6% of the population of the United States. FoodNet was expanded to selected counties in Maryland and New York in 1997. The goals of FoodNet are to describe the epidemiology of new and reemerging bacterial, parasitic, and viral foodborne pathogens; estimate the frequency and severity of foodborne diseases that occur in the United States per year; and determine how much foodborne illness results from eating specific foods, such as meat, poultry, and eggs.

Foodborne diseases are common; an estimated 6 to 33 million cases occur each year in the United States. Although most of these infections cause mild illness, severe infections and serious complications do occur. The public health challenges of foodborne diseases are changing rapidly; in recent years, new and reemerging foodborne pathogens have been described, and changes in food production have led to new food safety concerns. Foodborne diseases have been associated with many different foods, including some previously thought to be safe, such as eggs and fruit juice, both of which have transmitted *Salmonella* during recent outbreaks. Public health officials in the seven EIP sites are monitoring foodborne diseases, conducting epidemiologic and laboratory studies of these diseases, and responding to new challenges from these diseases. Information gained through this network will lead to new interventions and prevention strategies for addressing the public health problem of foodborne diseases.

Current “passive” surveillance systems rely upon reporting of foodborne diseases by clinical microbiology laboratories to state health departments, which in turn report to CDC. Although foodborne diseases are extremely common, only a fraction of them are routinely reported to CDC through these surveillance systems. Inadequate reporting results from a complex chain of events that must occur before a case is reported, and a break at any linkage along the chain results in a case not being reported (Figure). FoodNet is an “active” surveillance system, meaning public health officials frequently contact microbiology laboratory directors to find new cases of foodborne diseases and report these cases electronically to CDC. In addition, FoodNet is designed to monitor each of the events that occurs along the foodborne diseases pyramid and thereby allow more accurate and precise estimates and interpretation of the prevalence of foodborne diseases over time. Because most foodborne infections cause diarrheal illness, FoodNet focuses these efforts on persons who have a diarrheal illness.

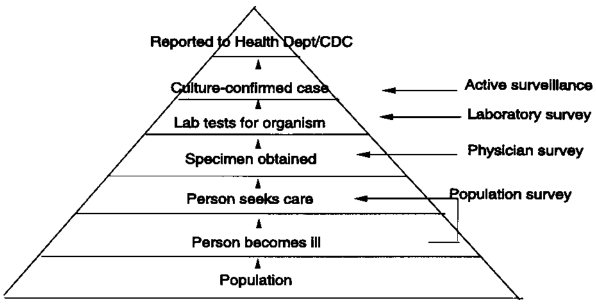

Figure. The prevalence of illness pyramid. Passive surveillance data represent only the tip of the iceberg. For a bacterial infection to be included in the passive surveillance system, it must pass through the following steps: a person becomes ill with a diarrheal disease, the patient must go to a doctor, the doctor must order a bacterial stool culture, the assigned microbiology laboratory must culture for this organism and report the infection to the state health department, and the state health department in turn must report the infection to CDC. This passive surveillance system is the means by which the number of cases of foodborne illness is currently determined at CDC; if any step does not occur, foodborne illness is not reported. FoodNet is designed to collect information along each step of this pyramid.

### FoodNet Components

### Active Laboratory-Based Surveillance

The core of FoodNet is population-based active surveillance at over 300 clinical microbiology laboratories that test stool samples in the seven participating sites. In active surveillance, the laboratories in the catchment areas are contacted regularly by collaborative FoodNet investigators to collect information on all laboratory-confirmed cases of diarrheal illness. Since January 1996, information has been collected on every laboratory-diagnosed case of *Salmonella*, *Shigella*, *Campylobacter*, *Escherichia coli* O157, *Listeria*, *Yersinia*, and *Vibrio* infection among residents of the catchment areas of the five original sites; this information is transmitted electronically to CDC. The result is a comprehensive and timely database of foodborne illness in a well-defined population.

### Survey of Clinical Laboratories

In October 1995, collaborative FoodNet investigators conducted a baseline laboratory survey of all microbiology laboratories in the five original catchment areas to determine which pathogens are included in routine bacterial stool cultures, which tests must be specifically requested by the physician, and what specific techniques are used to isolate the pathogens. A baseline survey will be conducted in the two new sites, and a follow-up survey to assess any recent changes in laboratory practices was conducted in the original sites in 1997. Practices in clinical laboratories have been found to vary; some laboratories look for a wider variety of bacteria than others. The methods used to collect and examine specimens are being investigated because these can influence whether the laboratory finds disease-causing bacteria.

### Survey of Physicians

To obtain information on physician stool-culturing practices, collaborative FoodNet investigators mailed a survey questionnaire to 5,000 physicians during 1996. Analysis of these data is ongoing. Because laboratories test stool specimens from a patient only upon the request of a physician or other health-care provider, it is important to measure how often and under what circumstances physicians order these tests. As changes occur in the way health care is provided in the United States, stool-culturing practices may also change. The practices of physicians who send stool samples to laboratories within the catchment areas will be monitored by surveys and validation studies.

### Survey of the Population

Collaborative FoodNet investigators contact randomly selected residents of a catchment area and ask whether the person has had a recent diarrheal illness, whether the person sought treatment for the illness, and whether the person had consumed certain foods known to have caused outbreaks of foodborne illness. During 1996, 750 residents of the catchment areas were interviewed by telephone each month (9,000/year). Because many who become ill with diarrhea do not see a physician, little is known about the number of cases of diarrhea in the general population and how often persons with diarrhea seek medical care. The population survey is an essential part of active surveillance for foodborne illness because it allows for an estimate of the population who seeks medical care when affected by diarrheal illness.

### Case-Control Studies

In 1996, the FoodNet began case-control studies of *E. coli* O157 and *Salmonella* serogroup B and D infections. More than 60% of *Salmonella* infections in the United States are caused by serogroup B and D *Salmonella*. These large case-control studies will provide new and more precise information about which food items or other exposures may cause these diseases. To allow the most precise classification of the isolates from the patients in these studies, the *Salmonella* and *E. coli* O157:H7 laboratory specimens from these patients are sent from FoodNet sites to CDC for further study, including antibiotic resistance testing, phage typing, and molecular subtyping.

### Accomplishments

Since becoming operational on January 1, 1996, FoodNet has tracked the rates of foodborne diseases. Even in the first year of data collection, numerous interesting patterns and outbreaks were detected. Surprisingly high isolation rates for *Y. enterocolitica* in Georgia and *Campylobacter* in California were detected. An outbreak of *Salmonella* infections caused by contaminated alfalfa sprouts was detected in Oregon. Two outbreaks of *E. coli* O157:H7 infections were detected in Connecticut, one due to lettuce and one to apple cider. FoodNet has also provided the infrastructure for conducting active surveillance for new and reemerging diseases. When an association between bovine spongiform encephalopathy in cattle and variant–Creutzfeldt-Jakob disease in humans was suspected in the United Kingdom, EIP personnel conducted surveillance for this rare human disease. EIP personnel also collaborated in the investigation of a multistate outbreak of *Cyclospora* infections associated with consumption of raspberries from Guatemala.

### Future FoodNet Projects

Several projects in 1997 will focus on *Campylobacter*, including a case-control study to determine the risk factors for infection and determination of antibiotic resistance patterns among *Campylobacter* strains.

Collaborative FoodNet investigators will establish active surveillance for hemolytic uremic syndrome, a serious complication of *E. coli* O157:H7 infection.

**FoodNet Working Group***

*Frederick Angulo, David Swerdlow, Robert Tauxe, Patricia Griffin, Drew Voetsch, Thomas Boyce, Sudha Reddy, Mary Evans, Sam Yang (CDC); Duc Vugia, Ben Werner, Kevin Reilly (California Department of Health Services); Sue Shallow, Gretchen Rothrock, Pam Daily, Felicia Chi (California EIP); Paul Blake, Jane Koehler (Georgia Department of Human Resources); Monica Farley, Wendy Baughman, Molly Bardsley, Suzanne Segler, Shama Desai (Georgia EIP); James Hadler, Pat Mshar (Connecticut Department of Public Health); Ruthanne Marcus, Terry Fiorentino (Connecticut EIP); Michael Osterholm, Craig Hedberg, Jeff Bender, Julie Hogan, Valerie Deneen, Heidi Kassenborg (Minnesota Department of Health); Paul Cieslak, John Townes, Beletshachew Shiferaw, Maureen Cassidy, Theresa McGivern, Regina Stanton (Oregon Health Division); Diane Dwyer, Peggy Pass (Maryland Department of Health and Mental Hygiene); and Dale Morse, Julia Kiehlbauch, Hwa-Gan Chang, Cathy Stone (New York Department of Health); I.Kaye Wachsmuth, Jill Hollingsworth, Peggy Nunnery, Art Baker, Phyllis Sparling (USDA-FSIS); Ken Falci, Bing Garthright, Sean Altekruse (FDA-CFSAN).

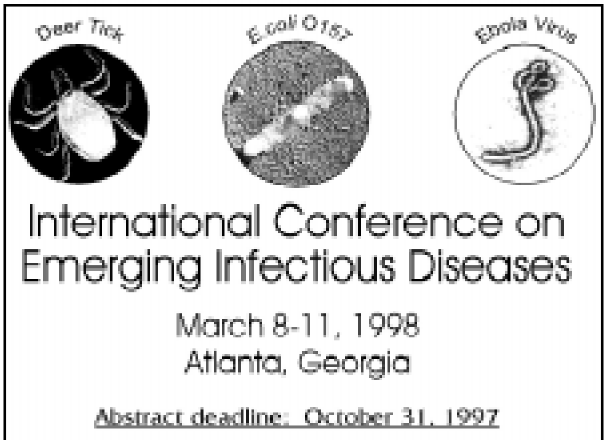

The Centers for Disease Control and Prevention, the Council of State and Territorial Epidemiologists, the American Society for Microbiology, and the National Foundation for CDC, along with more than 50 agencies and organizations are cosponsoring the 1998 International Conference on Emerging Infectious Diseases. This conference will offer new opportunities for education, collaboration, and partnership with colleagues worldwide to explore the most current research, surveillance, and prevention and control programs addressing all aspects of emerging infectious diseases. Attendance is limited to 2,500 participants.

The meeting will consist of general and plenary sessions, symposia, and roundtables with invited speakers, presentations on emerging infections activities, oral and poster presentations based on submission of an accepted abstract, and exhibits. Conference topics will include work on surveillance, epidemiology, research, communication, training, and prevention and control of emerging infectious diseases, as well as emergency preparedness and response.

Abstracts should address new, reemerging, and drug-resistant infectious diseases that affect human health. Deadline for abstract submission is October 31, 1997. Information on later submission of abstracts for consideration as late breakers is included in the program materials. For additional information, access announcements at www.cdc.gov/ncidod/EID/eid.htm or the National Center for Infectious Diseases (www.cdc.gov/ncidod/whatsnew.htm), send an e-mail to meetinginfo@asmusa.org, or call 202-942-9248. Proceedings of the conference will be published in the Emerging Infectious Diseases journal.

